# Oesophageal cancer magnitude and presentation in Ethiopia 2012–2017

**DOI:** 10.1371/journal.pone.0242807

**Published:** 2020-12-01

**Authors:** Abigiya Wondimagegnehu, Selamawit Hirpa, Samson Wakuma Abaya, Muluken Gizaw, Sefonias Getachew, Wondimu Ayele, Robel Yirgu, Tamiru Demeke, Berhe Dessalegn, Jilcha Diribi, Mirgissa Kaba, Mathewos Assefa, Ahmedin Jemal, Eva Johanna Kantelhardt, Adamu Addissie

**Affiliations:** 1 Department of Preventive Medicine, School of Public Health, College of Health Sciences, Addis Ababa University, Addis Ababa, Ethiopia; 2 Institute of Medical Epidemiology, Biostatistics and Informatics, Martin-Luther-University, Halle, Germany; 3 GINGER Research Fellow, Harvard T.H. Chan School of Public Health, Boston, MA, United States of America; 4 Department of Reproductive Health and Health Service Management, School of Public Health, Addis Ababa University, Addis Ababa, Ethiopia; 5 College of Medicine and Health Sciences, Adigrat University, Adigrat, Ethiopia; 6 Department of Oncology, Saint Paul's Hospital Millennium Medical College, Addis Ababa, Ethiopia; 7 Department of Oncology, School of Medicine, College of Health Sciences, Addis Ababa University, Addis Ababa, Ethiopia; 8 American Cancer Society, Atlanta, Georgia, United States of America; 9 Department of Gynecology, Martin-Luther-University, Halle, Germany; Howard University, UNITED STATES

## Abstract

The aim of this study was to assess the magnitude, socio-demographic, and clinical characteristics of oesophageal cancer patients in selected referral hospitals of Ethiopia. A retrospective document review was employed in ten referral hospitals in different regions of Ethiopia. A structured data extraction tool was used to extract data from clinical care records of all clinically and pathologically confirmed oesophageal cancer patients who were diagnosed and treated in those hospitals from 2012 to 2017. During the study period, a total of 777 oesophageal cancer cases were identified, and the median age of these patients was 55 years, with an interquartile range of 19. More than half (55.1%, n = 428) of the cases were males, and the majority of them were reported from Oromia (49.9%, n = 388) and Somali (25.9%, n = 202) regional states. The highest numbers of oesophageal cancer cases were recorded in 2016 (23.8%, n = 185), while the lowest were in 2012 (12.6%, n = 98). Eighty per cent of oesophageal cancer cases were diagnosed in later stages of the disease. More than one-fourth (27.0%, n = 210) of patients had surgical procedures where the majority (74.3%, n = 156) required insertion of a feeding tube followed by transhiatal oesophagectomy (10.9%, n = 23). Of the 118 patients for which there was histology data, squamous cell carcinoma (56.7%, n = 67) and adenocarcinoma (36.4%, n = 43) were the predominant histologic type. One-fourth (25.0%, n = 194) of the patients were alive, and more than two-thirds (71.7%, n = 557) of the patients’ current status was unknown at the time of the review. In these referral hospitals of Ethiopia, many oesophageal cancer patients presented during later stages of the disease and needed palliative care measures. The number of patients seen in Oromia and Somali hospitals by far exceeded hospitals of the other regions, thus postulating possibly unique risk factors in those geographic areas.

## Introduction

Oesophageal cancer is the leading cause of cancer-related mortality due to its subtle disease course and poor prognosis [1]. According to the GLOBOCAN 2018 report, approximately 572,034 new cases and 508,585 deaths from oesophageal cancer were estimated worldwide [2]. Oesophageal cancer incidence varies globally, with its highest across the ‘oesophageal cancer belt’, namely East and South African countries, and Asia [3, 4]. The two main types of oesophageal cancers are squamous cell carcinoma and adenocarcinoma [5]. Squamous cell carcinoma is more common in central, eastern, and southern parts of Africa, with the African oesophageal squamous cell carcinoma (ESCC) corridor stretching from the southern part of Sudan to the Eastern Cape Province of South Africa [6, 7]. Ethiopia is one of the countries along the oesophageal belt. A study from the largest referral hospital in Ethiopia indicated that oesophageal cancer was more common among patients from Arsi and Bale when compared to other regions [6].

There is no final consensus on the list of risk factors for oesophageal cancer, but in many studies, smoking and drinking alcohol showed a strong association with squamous cell carcinoma [8–12]. Exposure to silica, asbestos fibre, ionising radiation, polycyclic aromatic hydrocarbon, and pesticides were also observed to increase the risk [13–18]. In addition, the consumption of hot food and beverages was associated with an increased risk of ESCC [19–23].

Stage of cancer at diagnosis determines the disease prognosis. The overall oesophageal cancer survival rate is low because ESCC is often diagnosed late due to asymptomatic presentation during the early stage. In nearly 50% of the cases, the lesions metastasized by the time of diagnosis [24]. Delayed diagnosis is even more prominent in developing countries with limited access to cancer care. At one of the referral hospitals in Ethiopia, 56% of oesophageal cancer cases underwent an operation. Only 24% of those cases were suitable for esophagectomy. Thus, the postoperative mortality rate (28%) was also high [25]. Coupling early case identification with prompt treatment could improve patient survival and quality of life.

Despite its high prevalence, little is known about oesophageal cancer in Ethiopia. The few existing facility-based studies are inconclusive and do not provide a clear account of the disease at a national level. This study aims to generate a national estimate of the magnitude of oesophageal cancer, describe its clinical presentation, and identify the treatment outcomes in tertiary hospitals.

## Methods

### Study design and setting

The study team visited twelve selected hospitals in Ethiopia and reviewed the hospitals’ registry books to identify oesophageal cancer cases over the six years period (2012 to 2017). Out of these hospitals, data were collected from 10 referral hospitals located in seven regional states and one city administration: Amhara, Oromia, Southern Nations’ Nationality People Region (SNNPR), Tigray, Afar, Harari, Somali, and Dire Dawa. The largest referral hospital was selected for those regions that have more than one referral hospital. From Oromia regional state, Aira general, Goba referral, and Arsi University referral hospitals were purposefully selected since previous studies indicated that oesophageal cancer is common in those areas. Addis Ababa city administration was not included in this study because a similar study was conducted at Tikur Anbessa specialised hospital one year prior. Initially, we also planned to include two general hospitals from Benishangul and Gambella regional states. Despite the effort in reviewing the registry books, we were unable to find a single suspected or referred oesophageal cancer case. Therefore, no data was collected from these two sites, and they were not included in the analysis of the present study.

### Data sources and study population

Secondary data was collected from the aforementioned hospitals’ registry books and oesophageal cancer patients’ cards from 2012 to 2017. The inclusion criteria were all clinically and/or pathologically confirmed oesophageal cancer cases that were diagnosed and treated in those selected hospitals during the study period. Suspected cancer cases were included in the study because the majority of the hospitals did not have diagnostic techniques to confirm oesophageal cancer diagnosis. Data was not extracted from oesophageal cancer patients’ card with incomplete personal information such as age, sex, date of first diagnosis, and type of treatment received during data collection.

### Data collection tools and procedures

A structured data extraction tool was used to collect essential variables like socio-demographic factors (such as age, sex, marital status, level of education, religion, residence, occupation), and clinical characteristics of the patients (such as date of diagnosis, chief compliant at first presentation, stage and histologic type of cancer, type of treatment provided, current status and last date of follow up). Health professionals with bachelor degree and experience in data collection were recruited for data collection and supervision. They were provided with a one-day training covering the purpose of the study, questionnaire content, and field procedures. In addition, supervisors were trained in data quality control procedures and fieldwork coordination. At the end of the training, each data collector conducted pre-tests to familiarise him/herself with the data extraction tool.

### Data management and analysis

Various precaution measures were taken to ensure the quality of the data. Field supervisors and coordinators verified the completeness and consistency of the collected data at the end of the day during the data collection phase. The data were entered and cleaned using EpiData version 3.2 and transferred to SPSS version 23.0 for analysis. Descriptive statistics such as (percentages) and measures of central tendency and dispersion were computed to describe oesophageal cancer cases by person, place, and time. All clinical characteristics of oesophageal cancer patients were presented in the form of frequency tables and figures.

### Ethics approval and consent to participate

Ethical clearance of this study was obtained from the Research Ethics Committee of the School of Public Health and Institutional Review Board of the College of Health Science, Addis Ababa University.

## Results

### Socio-demographic characteristics

The median age of oesophageal cancer patients was 55 years with an interquartile range (IQR) of 19 years. More than half (55.1%, n = 428) of them were male, 342 (44.1%) were Muslim, and 113 (14.5%) were Orthodox Christians. However, religion was not recorded for 305 (39.3%) of the patients. The patients’ cards showed that 137 (17.6%) were illiterate, 45 (8.4%) attained primary education, and 35 (4.5%) achieved secondary education or beyond. Half (50.5%, n = 392) of the oesophageal cancer patients were married while marital status was not recorded for more than one-third (38.5%) of the patients. Occupational status was not documented for most (68.0%, n = 528) of the study participants. However, 103(13.3%), 99 (12.7%), and 12 (1.2%) of the patients were housewives, farmers, and factory workers, respectively (Table 1).

**Table 1:** Socio-demographic characteristics of oesophageal cancer patients in selected hospitals, Ethiopia, 2018.

Socio-demographic characteristics	Number	Per cent (%)
**Median age (IQR)**	55 (19)	
**Sex**		
Male	428	55.1
Female	342	44.0
Unknown	7	0.9
**Name of the hospitals**		
Aira GH	161	20.7
Arsi University	2	0.3
Ayder RH	69	8.9
Dilchora RH	21	2.7
Dubti RH	30	3.9
Goba RH	119	15.3
Gondar University RH	10	1.3
Hawassa University RH	160	20.6
Hiwot Fana Specialized H	6	0.8
Karamara RH	199	25.6
**Region**		
Afar	34	4.4
Amhara	16	2.1
Dire Dawa	13	1.7
Harari	1	0.1
Oromia	388	49.9
SNNPR	64	8.2
Somali	202	26.0
Tigray	59	7.6
**Religion**		
Orthodox	113	14.5
Protestant	14	1.8
Muslim	342	44.0
Catholic	3	0.4
Unknown	305	39.3
**Education**		
Illiterate	137	17.6
Read and write	65	8.4
Primary education	21	2.7
Secondary and above	35	4.5
Unknown	519	66.8
**Marital status**		
Single	19	2.4
Married	392	50.5
Divorced/separated	42	5.4
Widowed	29	3.7
Unknown	295	38.0
**Occupation**		
Housewife	103	13.3
Farmer	99	12.7
Civil servant at office	35	4.5
Factory worker	12	1.5
Unknown	528	68.0

**IQR**: interquartile region, **GH**: General Hospital, **RH**: Referral Hospital, **SNNPR**: Southern Nations, Nationalities, and People’s Region.

**Fig 1:**
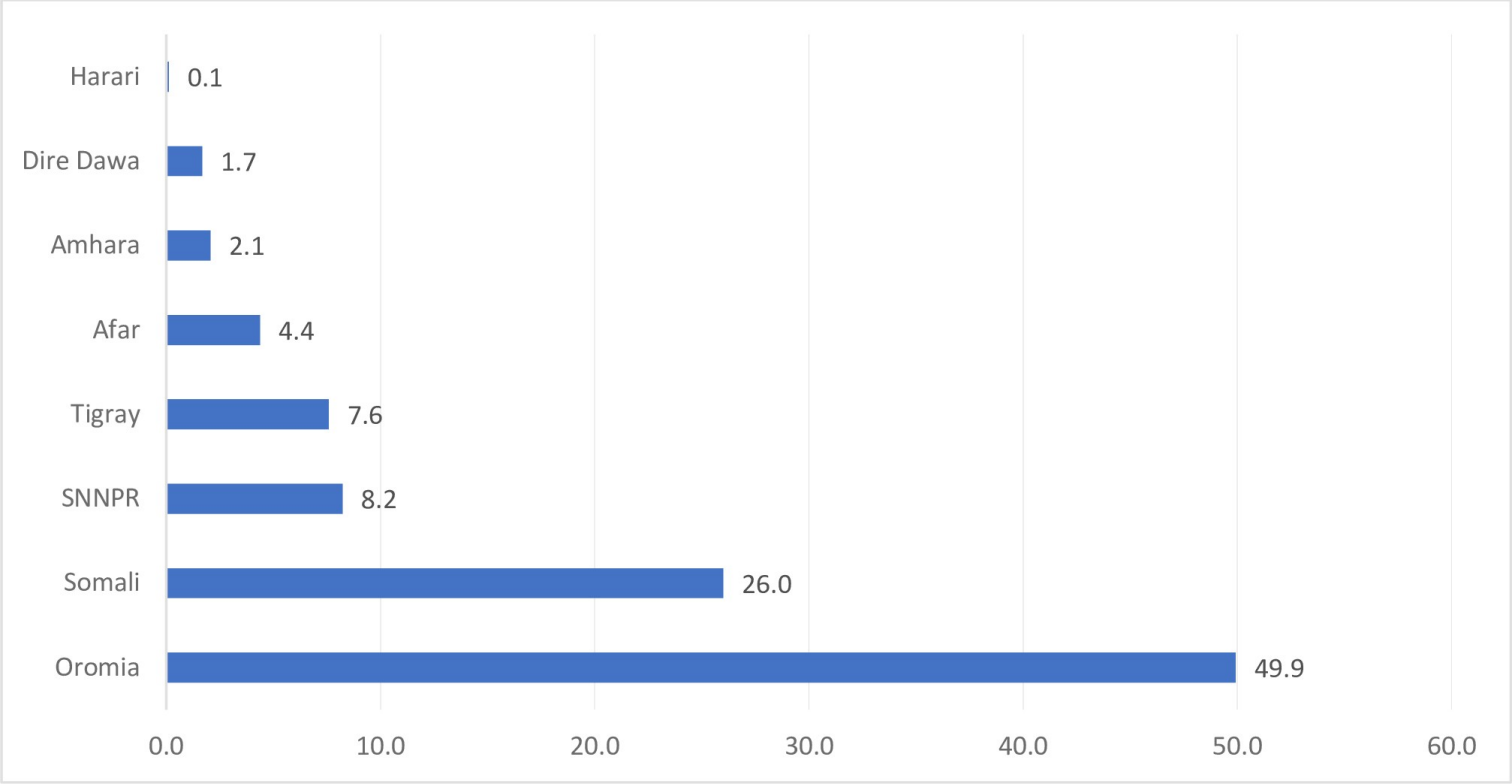
Distribution of oesophageal cancer patients. Patients in the different regions of Ethiopia from 2012 to 2017.

Three-fourths (75.9%, n = 590) of the oesophageal cases were reported from Oromia and Somali regions with a total of 388 (49.9%) and 202 (26.0%) cases, respectively. The remaining one-fifth of the cases were reported from the SNNPR 64 (8.2%), Tigray 59 (7.6%), and Afar 34 (4.4%) regions (Fig 1).

**Fig 2:**
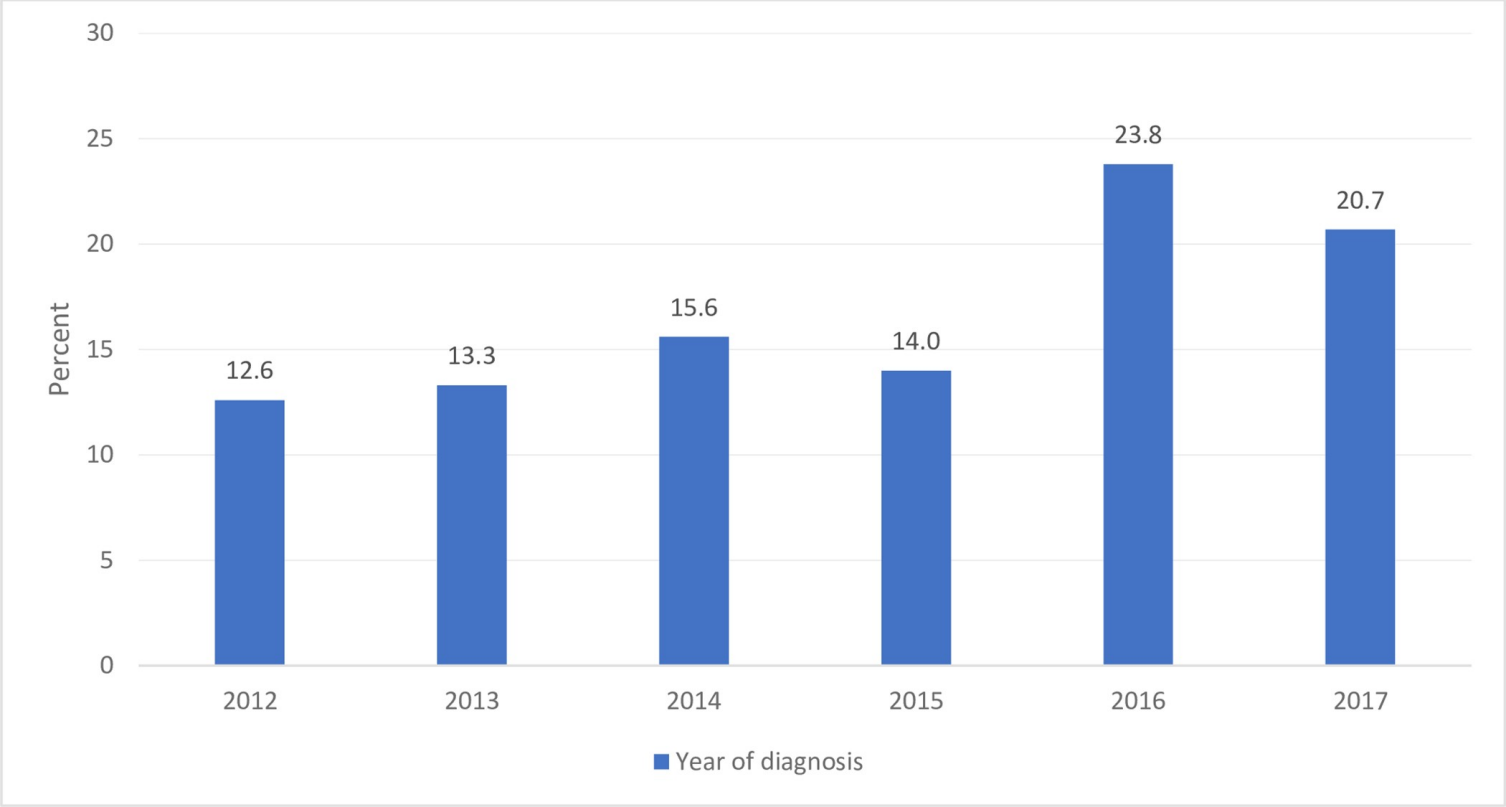
Time trends. Oesophageal cancer cases in in Ethiopia from 2012 to 2017.

With regard to the magnitude of oesophageal cancer over the past six years, the highest number of oesophageal cases was reported in 2016 with a total of 185 (23.8%) cases, followed by 161 (20.7%) cases in 2017, and the lowest was reported in 2012 with a total of 98 (12.6%) cases (Fig 2).

### Medical characteristics of oesophageal cancer patients

Of the total patients diagnosed and treated during the study period, 692 (89.1%) presented to the hospital with a chief complaint of difficulty swallowing, followed by 374 (49%) of weight loss, and 233 (30%) of heartburn. In this study, only 98 (12.6%) of oesophageal cases had comorbid illnesses such as hypertension 75 (76.5%) and diabetics mellitus 43 (43.3%) (Table 2).

**Table 2:** Medical and clinical characteristics of oesophageal cancer patients in selected hospitals, Ethiopia, 2018.

Variables	Frequency	Percentage (%)
**Chief complaints (n = 777)**		
Difficulty swallowing	692	89.1
Weight loss	374	48.1
Heartburn	233	30.0
Vomiting	327	42.1
Cough	122	15.7
Chest pain	130	16.7
Hoarseness of voice	75	9.7
**Comorbidities (n = 99)**		
Hypertension	75	76.5
Diabetes Mellites	43	43.3
HIV	15	15.2
Others*	2	2.0
**Presence of pertinent physical examination (n = 777)**		
Yes	238	30.6
No	60	7.7
Unknown	479	61.6
**Physical examination finding**		
Respiratory finding	124	52.1
Neck mass	92	38.6
Lymphadenopathy	76	31.9
Abdominal mass	56	23.5
**Methods of diagnosis (n = 777)**		
Endoscopy	316	40.7
Chest X ray	171	22.0
Barium swallow	134	17.2
Biopsy	129	16.6
CT/MRI	79	10.2
Abdomino-pelvic ultrasound	15	1.9
**Histologic type (n = 316)**		
Squamous cell carcinoma	67	67.0
Adenocarcinoma	43	43.0
Adenosquamous cell carcinoma	8	8.0
Unknown	198	198.0
**Histologic Grading (n = 316)**		
Well-differentiated	11	3.5
Moderately differentiated	53	16.8
Poorly differentiated	36	11.4
Undifferentiated/ Unspecified	18	5.6
Unknown	198	62.7
**Endoscopy findings (n = 316)**		
Mass	209	66.1
Ulcer	106	33.5
Stricture	93	29.4
Polyp	44	13.9
Oesophageal web	12	3.8
Esophagitis	45	14.2
GERD	39	12.3
Other**	4	1.2
**Site of mass in endoscopy (n = 316)**		
Cervical	13	4.1
Upper thoracic	37	11.7
Middle thoracic	98	31.0
Lower thoracic	75	23.7
G-E junction/cardia	37	11.7
Unknown	56	17.7
**Stage of cancer**		
Stage I	1	0.8
Sage II	24	19.5
Stage III	84	68.3
Stage IV	14	11.4

“NB” Other*: Anaemia and TB, GERD: Gastroesophageal Reflex Disease, Other** Bari at oesophagus and gastropathy

Of the total number of patients included in this study, nearly two-thirds (61/6%, n = 479) were missing physical examination results. The patients’ cards revealed that respiratory findings (52.1%, n = 124) and neck mass (38.6%, n = 92) were the most common observations among the physical exams. Of the diagnostic methods, more than one-third (40.7%, n = 316) of oesophageal cancer patients were diagnosed using endoscopy. Out of the total number of patients who had an endoscopic diagnosis, we obtained histopathological results for 118 (37.3%). Among those 118 results analysed for histopathological subtype, squamous cell carcinoma accounts (56.7%, n = 67) were the most prevalent, followed by adenocarcinoma (36.4%, n = 43). Of the patients who underwent endoscopy, the site of mass was located in two-thirds of them (66.1%, n = 209). Oesophageal cancers were found in the middle thoracic region in 98 (31%) patients, followed by the lower thoracic region with 75 (23.7%) patients. From the total 123 patients whose cancer stage was recorded, 98 (80%) of them were in stage III and IV during their first presentation (Table 2).

### Treatment pattern and outcome

The patients’ cards revealed that 210 (27.0%) patients had surgical intervention. The most common type of surgery performed was a feeding tube insertion in 156 (74.3%) patients, followed by transhiatal oesophagectomy in 23 (10.9%), and thoracotomy/laparotomy in 19 (9.0%). However, nearly one-third (31.0%, n = 246) of the patients’ files did not describe whether or not they had surgery. Diaphragm (2.2%, n = 17), pericardium (1.2%, n = 9), and pleura (1.0%, n = 8) were the most frequently reported adjacent structures involved based on the intraoperative findings and imaging results in the patients’ profile. However, it was found that most (92.4%, n = 718) of the patients’ profiles had no information related to adjacent site involvement.

Out of the 777 patients’ charts that were reviewed, only 39 (5.0%) patients had received chemotherapy. However, most of the patients (71.9%, n = 557) had no information on chemotherapy status. The most prescribed regimen was cisplatin/5-FU in 22 (56.4%) patients, and cisplatin/paclitaxel in 15 (38.4%). Out of the patients in chemotherapy follow-up, 20 (51.2%) had taken between two and five treatment cycles, whereas 15 (38.5%) completed six cycles of treatment. From all cases reviewed, 365 (47.0%) had 2–5 previous visits to the hospital, and 260 (33.5%) of patients only visited once. We found that 194 (25.0%) of the patients were alive, 26 (3.3%) had died, and the current status was unknown for more than two-thirds (71.7%, n = 557) of the patients at the time of the review (Table 3).

**Table 3:** Treatment pattern and outcome of Oesophageal cancer patients in selected hospitals, Ethiopia, 2018.

Characteristics	Frequency	Percentage
**Surgery completed on the patients (n = 777)**		
Yes	210	27.0
No	321	41.0
Unknown	246	31.0
**Surgery types performed (n = 210)**		
Feeding tube	156	74.3
Transthoracic esophagostomy (TTE)	13	6.2
Transhiatal esophagectomy (THE)	23	10.9
Thoracotomy/Laparotomy	19	9.0
**Adjacent structure involved (n = 777)**		
No involvement	21	2.7
Pleura	8	1.0
Pericardium	9	1.2
Diaphragm	17	2.2
Stomach	2	0.3
Laryngeal nerve	2	0.3
Others*	4	0.4
Unknown	718	92.4
**Chemotherapy given (n = 775)**		
Yes	39	5.0
No	179	23.1
Unknown	557	71.9
**Regimen of chemotherapy given (n = 39)**		
Cisplatin + 5-FU	22	56.4
Cisplatin + paclitaxel	15	38.4
Carboplatin + paclitaxel	1	2.6
Cisplatin + irinotecan	1	2.6
**Cycle of chemotherapy (n = 39)**		
1	4	10.3
2–3	10	25.6
4–5	10	25.6
Completed 6 cycles	15	38.5
**Patients number of visits to hospital (n = 777)**		
1	260	33.5
2–5	365	47.0
6–10	12	1.5
Unknown	140	18.0
**Outcome of patients (n = 777)**		
Alive	194	25.0
Died	26	3.3
Unknown	557	71.7

“NB”. Other*: Aorta/subclavian artery, Larynx/tracheal/bronchus, Thyroid, Vertebral body

## Discussion

A total of 777 oesophageal cancer patients’ charts, diagnosed between 2012 and 2017, were reviewed for this study. Out of those reviewed 55% were males, with a median age of 55 years. Difficulty of swallowing (dysphagia) was the main complaint in majority (90.6%) of patients with oesophageal cancer followed by weight loss in 48.1%, of cases during hospital presentation. From the analysed histopathological findings 56.7% were squamous cell type of carcinoma. Half of oesophageal cancer cases were reported from Oromia region and at the time of presentation 80% of patients were diagnosed in the late stage.

In most cases, oesophageal cancer is three to four times more common in males than females [25, 26]. A systematic review conducted in sub-Saharan African countries showed that male predominate oesophageal cancer cases with ratio of 2:1 [26]. A systematic review and a meta-analysis conducted in 36 African countries revealed that being male was a risk factor for oesophageal cancer (1.7; 95% Confidence Interval: 1.4, 2.0) [27]. In contrast, a study conducted in Sudan reported a 1:1.8 male to female ratio of oesophageal cancer cases [28]. In the present study, the female to male ratio was 1:1.3, which was not as high as studies conducted in many developed countries and other African countries. The difference could be that in most Western countries, the risk factors for oesophageal cancer are tobacco smoking and alcohol. Meanwhile, the prevalence of cigarette smoking and alcohol consumption were low among oesophageal cancer patients in Ethiopia, representing 5% and 2% of the cases, respectively [29]. The demographic and health survey Ethiopia 2016 reports rather low “ever alcohol consumption” prevalence for Oromia and Somali regions (14.4/25.5 and 0.3/1.0% for females/males). Oromia shows the highest proportion of women and men who drink nearly every day in the past week (11.3 and 25.3%) compared to lower proportions in most other regions [30]. This could contribute to high numbers of oesophageal cancer patients in Oromia. In Oromia 4.4%, in Somali 19.1% of all men smoke daily, less than 1% of women smoke in both regions. These findings may explain higher number of oesophageal cancer patients from Somali region compared to other regions. Still the overall prevalence of alcohol consumption and smoking is lower compared to other regions of the world where far less oesophageal cancer patients are seen. Detailed information about possible risk factors such as hot porridge or hot tea consumption is still lacking. A reason why a high proportion of men were diagnosed with oesophageal cancer in our study could be related to better health-seeking behaviour of men compared to women.

This study indicated that the median age of the study participants was 55 years. A systematic review in sub-Sahara Africa indicated that the median age of oesophageal cancer patients was 59 years [26]. Another study conducted in Ghana indicated the mean age of the oesophageal cancer patients was 57.8 years [31]. These finding can be explained by the fact that the chance of getting oesophageal cancer cases increase with age. However, the median age of oesophageal cancer patients can generally vary from country to country since it highly depends on the underlying population structure.

In the present study, squamous cell carcinoma was identified in 67 (56.7%) patients. This finding is consistent with studies conducted in Kenya, North Sudan, Uganda, which all showed a higher prevalence of ESCC as compared to adenocarcinoma [32, 33]. A systematic review showed that the squamous cell subtype is the predominant carcinoma in Africa [27]. Generally, from the two common subtypes of oesophageal cancer, squamous cell carcinoma is the most common worldwide and is especially common among black men [34].

In this study, almost 50% of oesophageal cancer cases originated from the Oromia region, specifically from Bale, Arsi, and Wellega. Ethiopia is one of the oesophageal cancer belt countries; Arsi and Bale are known hot spot areas [6]. A study done at Tikur Anbessa hospital found most oesophageal cancer cases were higher among patients from Arsi and Bale areas as compared to other regions [35]. In Ethiopia, most oesophageal cancer patients come from rural areas, mainly from the southern and eastern parts of Ethiopia. Most of the risk factors indicated for oesophageal cancer, such as smoking and consuming alcohol, were not present in most oesophageal cancer cases in Ethiopia [29]. According to a pilot case control study in Addis Ababa, higher proportion of EC cases (36%) than controls (26%) were ever qat (Catha edulis) chewers but no excess risk of EC in association with ever use of qat was observed after adjusting for confounding factors [36]. This could indicate potential dietary factors such as porridge and kocho as risk factor for oesophageal cancer [36]. This could explain why most cases are from a similar region, and further study is needed to identify risk factors of oesophageal cancer in these areas.

Of the patients who underwent endoscopy, the site of mass was located in the majority of the patients (66.1%, n = 209). With regard to the site of the mass, oesophageal cancers were found in the middle thoracic region in 98 (31%). This finding is consistent with the study completed at Tikur Anbessa Hospital, where the middle lower part was the site for the mass in 49% of oesophageal cancer cases [32]. The approximate anatomical distribution of tumours within the oesophagus is <20% in the upper third, 30–70% in the middle third, and 20–50% in the lower third [37, 38]. The middle third of the oesophagus is the most common site for squamous cell carcinoma, and the lower third is the most common site for adenocarcinoma [3, 39–41]. Contrary to this, a study in Ghana showed that in 84.9% of cases, the mass was found at the distal third part [32].

Of the total patients seen during the study period, 692 (90.6%) presented to the hospital with a complaint of difficulty swallowing, followed by 374 (49%) who showed weight loss. Studies in different parts of Africa, including Ethiopia, revealed that most patients come to health facilities with difficulty of swallowing and weight loss [3, 39]. The disease is asymptomatic during the early stages, and most patients seek health care after experiencing an increase in clinical manifestation. This is also true for other types of cancers, especially in countries where awareness of and access to health care is low.

From the patient whose cancer stage was recorded, 98 (80%) of the patients were in stage III and IV during their first-time presentation. This finding is similar to the study in Kenya, where 70–80% of the patients were diagnosed in later stages [27]. This could be because most oesophageal cancer were asymptomatic during the early stages and diagnosed late when the outcome of the treatment is poor [34]. A review of data from the six countries indicated that more than 50% of oesophageal cancer patients come to the health facilities when they have metastatic disease [41]. In Ethiopia, patients diagnosed at Black Lion hospital also revealed the same problem. A five-year review of the oesophageal cancer showed that 56% of the cases were underwent surgical procedures, and only 24% were suitable for transhiatal esophagectomy, and the mortality after operation was 28% [35].

### Strength and limitation of the study

In Ethiopia, few studies have been conducted on oesophageal cancer, and most of the studies are based on data from one hospital. This study was conducted across ten hospitals located among six regions, and one city administration in Ethiopia. The results from this study can provide a better picture of the magnitude of oesophageal cancer in major hospitals in the country. One of the limitations of this study was the inclusion of suspected cancer cases because of unavailability of diagnostic techniques to confirm cases in the majority of the hospitals included in this study. This resulted in lack of uniformity of the diagnostic methods used. The other limitation is having high number of missing data from the medical record which might compromise the representativeness of the findings. Due to the retrospective nature of the study, we were unable to obtain information on important risk factors such as smoking or alcohol.

## Conclusions

Since most of the oesophageal cancer cases were diagnosed late, palliative treatment options such as oesophageal stent implantation are urgently needed. This applies especially for the regions (such as Oromia) where high numbers of patients are admitted to hospitals. More public awareness could possibly lead to earlier detection and higher cure rates even though the disease has high fatality rates even in higher resource settings.

### Recommendation

Further research is required to assess the risk factors for oesophageal cancer in the Oromia region, where the disease is more prevalent. It is also recommended to design mechanism for early detection and surveillance of precancerous lesions like in Barrett’s Oesophagus and expand therapeutic options including palliative care. An improved data recording system should be implemented in all hospitals in Ethiopia.
